# Injectable microspheres filled with copper-containing bioactive glass improve articular cartilage healing by regulating inflammation and recruiting stem cells

**DOI:** 10.1093/rb/rbae142

**Published:** 2024-12-17

**Authors:** Hua Gao, Eryu Ning, Xiaoyu Zhang, Zhiqiang Shao, Dan Hu, Lang Bai, Hui Che, Yuefeng Hao

**Affiliations:** Orthopedics and Sports Medicine Center, The Affiliated Suzhou Hospital of Nanjing Medical University, Suzhou Municipal Hospital, Gusu School, Nanjing Medical University, Suzhou 215008, P. R. China; Orthopedics and Sports Medicine Center, The Affiliated Suzhou Hospital of Nanjing Medical University, Suzhou Municipal Hospital, Gusu School, Nanjing Medical University, Suzhou 215008, P. R. China; Orthopedics and Sports Medicine Center, The Affiliated Suzhou Hospital of Nanjing Medical University, Suzhou Municipal Hospital, Gusu School, Nanjing Medical University, Suzhou 215008, P. R. China; Orthopedics and Sports Medicine Center, The Affiliated Suzhou Hospital of Nanjing Medical University, Suzhou Municipal Hospital, Gusu School, Nanjing Medical University, Suzhou 215008, P. R. China; Orthopedics and Sports Medicine Center, The Affiliated Suzhou Hospital of Nanjing Medical University, Suzhou Municipal Hospital, Gusu School, Nanjing Medical University, Suzhou 215008, P. R. China; Orthopedics and Sports Medicine Center, The Affiliated Suzhou Hospital of Nanjing Medical University, Suzhou Municipal Hospital, Gusu School, Nanjing Medical University, Suzhou 215008, P. R. China; Orthopedics and Sports Medicine Center, The Affiliated Suzhou Hospital of Nanjing Medical University, Suzhou Municipal Hospital, Gusu School, Nanjing Medical University, Suzhou 215008, P. R. China; Orthopedics and Sports Medicine Center, The Affiliated Suzhou Hospital of Nanjing Medical University, Suzhou Municipal Hospital, Gusu School, Nanjing Medical University, Suzhou 215008, P. R. China

**Keywords:** osteoarthritis, microspheres, bioactive glass, macrophages

## Abstract

Osteoarthritis (OA) is a frequent chronic illness in orthopedics that poses a major hazard to patient health. *In situ* cell therapy is emerging as a therapeutic option, but its efficacy is influenced by both the inflammatory milieu and the amount of stem cells, limiting its use. In this study, we designed a novel injectable porous microsphere (PM) based on microfluidic technology that can support *in situ* mesenchymal stem cells (MSCs) therapy by combining polylactic–glycolic acid copolymer, kartogenin, polydopamine, stromal cell-derived factor-1, and copper-doped bioactive glass (CuBG). The *ex vivo* tests demonstrated that PMs@CuBG microspheres were biocompatible and facilitated the transformation of synovial macrophages from pro-inflammatory M1 to anti-inflammatory M2 phenotypes by releasing CuBG to reduce joint inflammation. At the same time, the microspheres are able to recruit MSCs into the joint cavity and encourage their differentiation into chondrocytes, thereby treating articular cartilage injury. The *in vivo* rat experimental results show that intra-articular injection of PMs@CuBG in rats with OA improves OARSI scores, aggrecan content and the ratio of col-2α-positive cells, indicating a reparative effect on damaged cartilage within the joint. As a result, PMs@CuBG microspheres are predicted to provide a novel and successful approach to *in situ* cell therapy for OA.

## Graphical abstract

**Figure:**
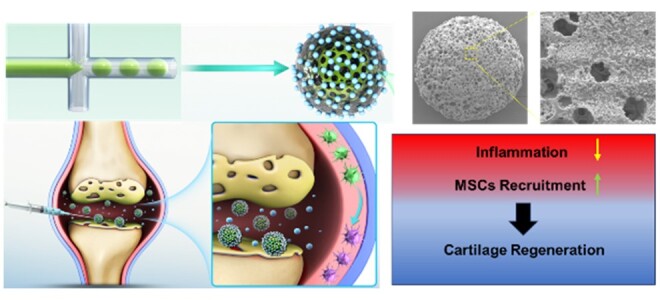

## Introduction

Osteoarthritis (OA) is a prevalent degenerative joint condition affecting almost 7% of the global population and represents a significant contributor to disability in older adults. OA has become more common in recent years as a result of an aging and obese population, and the health risks associated with it have increased [1]. OA causes alterations in tissues such as cartilage, synovium, subchondral bone, and ligaments and the pathophysiology is not entirely understood. The major hallmark of OA is articular cartilage degeneration. Synovial inflammation mediated by M1-type macrophages is regarded as one of the causes of OA progression [2]. Currently, non-surgical treatment for early- to mid-stage OA is based on the use of nonsteroidal anti-inflammatory medicines, which show little efficacy in slowing disease development [3]. In recent years, there have been numerous studies on the treatment of OA with tissue-engineered biomaterials and mesenchymal stem cell (MSC) therapy [4–7]. Tissue engineering has yielded some benefits in modulating synovial macrophage inflammation and combining cell therapies [8–10]. However, to implement *in situ* cell treatment, the following difficulties must still be addressed: (i) creating an environment in which recruited cells may attach and grow; (ii) minimizing the impact of joint inflammation on cell therapy. As a result, novel biomaterials that can reduce inflammation while also recruiting MSCs *in situ* must be investigated.

Bioactive glass (BG) is a type of multi-component, metal oxide composite with a SiO_2_ and CaO network structure as the major matrix. The cationic breakdown products can bind to the tissue surface and trigger various physiological responses, resulting in a variety of possible bioactivities [11, 12]. Copper, an essential trace element, has been found to guide the shift of macrophage polarization from pro-inflammatory M1 to anti-inflammatory M2 in physiological conditions [13]. Copper-doped BG (CuBG) is likely to reduce joint inflammation by controlling macrophage polarization, making it a promising candidate for cell therapy [14]. Furthermore, CuBG inhibits cartilage breakdown while increasing chondrocyte development, which contributes to the prevention of OA progression [15, 16].

Polylactic–glycolic acid (PLGA) copolymer is a material with excellent modulability and mechanical strength [7]. Our group previously used PLGA to create microspheres with appropriate pore sizes to allow cells to pass through and for kartogenin (KGN) to enter, which can promote MSCs differentiation into chondrocytes [17, 18]. However, PLGA microspheres make it challenging to transport significant volumes of water-soluble medicines. To address this issue, we used polydopamine (PDA) to create film-coated porous microspheres (PMs) [19, 20]. Because of the PDA coating’s encapsulation, CuBG could be attached to the microspheres via hydrogen bonding, resulting in significant microsphere loading [21]. Finally, stromal cell-derived factor-1 (SDF-1) was attached as a medication for intra-articular recruitment of MSCs, thereby promoting the activity of cells *in situ* [22, 23].

**Figure 1.**
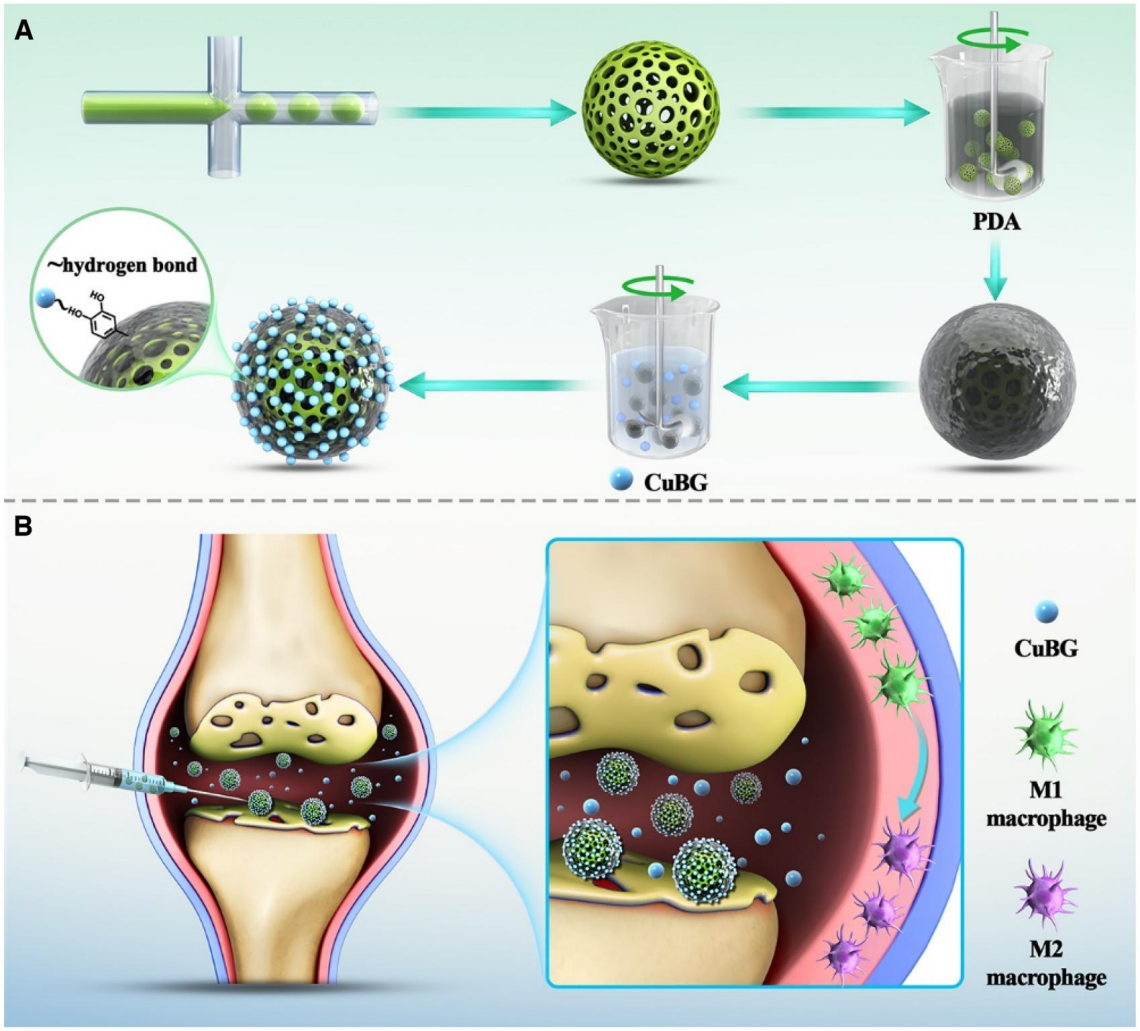
(**A**) Preparation of PMs@CuBG. (**B**) Schematic illustration on treatment of osteoarthritis with PMs@CuBG.

Here, we designed a microsphere made of PLGA containing KGN with PDA and SDF-1 on the outer surface to generate multifunctional PMs [18]. These were then coupled with CuBG to form PMs@CuBG. We hypothesized that, after being injected into the joint cavity, the microspheres would release the surface-loaded CuBG and SDF-1, thereby encouraging synovial macrophages to polarize to the M2 phenotype, regulating the joint's inflammatory milieu, and recruiting MSCs. KGN would then be gradually released from the microspheres, causing MSCs to develop into chondrocytes and stimulating cartilage-related gene expression (Figure 1). If effective, these microspheres would be able to heal articular cartilage damage and cure OA.

## Materials and methods

### Preparation of CuBG

CuCl_2_-2H_2_O (≥99.99%, Sigma-Aldrich) was dissolved in 50 ml of deionized water and heated to 80°C with magnetic stirring for 1 h. An aqueous solution of l-ascorbic acid (≥99%, Sigma-Aldrich) was added dropwise to the CuCl_2_ solution. The reaction was then stirred at 80°C for 24 h. The combination was then centrifuged at 7000 rpm for 20 min, and the supernatant containing the Cu/ascorbic acid complex suspension was refrigerated (4°C) for later use. MBGNs (85% SiO_2_ and 15% CaO, mol%) were created using the microemulsion-assisted sol–gel technique. 8 ml ethyl acetate was dissolved in 26 ml aqueous CTAB (20% w/v) and agitated for 30 min. Then 5.6 ml of ammonia was added, and stirring continued for 30 min. TEOS (2.88 ml) and 1.83 g of Ca(NO_3_)_2_ were added consecutively. To synthesize CuBG, 7 ml of the Cu/ascorbic acid combination was added to the mixture. After stirring for another 4 h and centrifuging for 20 min, the mixture was washed three times with deionized water/ethanol and dried overnight at 60°C. Finally, the powder was calcined at 700°C for 4 h, with a heating rate of 2°C/min. The final synthesized compounds were named CuBG.

### Preparation of microspheres

Drawing upon the methodologies previously employed by our research group, we utilized a microfluidic system to fabricate microspheres via a W/O/W (water/oil/water) emulsification technique. Specifically, we prepared solutions of 2% wt PLGA in dichloromethane, 7.5% wt gelatin in aqueous solution, and 1% PVA. The 2% wt PLGA in dichloromethane was blended with KGN at a mass ratio of 9:1, followed by mixing with the gelatin solution at a ratio of 3:1 (PLGA–KGN mixture to gelatin mixture). This blend was subjected to ultrasonic emulsification for 3 min (duty cycle 40%) to yield the inner phase. A 1% PVA solution served as the outer phase. The inner and outer phases were dispensed at a flow rate ratio of 1:40 through a capillary tube, collecting the microspheres which were then left to stir overnight. On the following day, they were subjected to a water bath at 40°C and filtered using a sieve with a pore size of 100 μm, repeating this process three times to collect the microspheres. Based on the previously established KGN standard curve (Supplementary Figure S5), a UV spectrophotometer (Beckman, Fullerton, CA, USA) was used to determine the amount of KGN added to the sample based on the absorbance at 287.4 nm using the following formula: entrapment efficiency (EE) = [(total amount of KGN added−amount of KGN free after inclusion)/total amount of KGN added] × 100% to calculate the KGN EE. Subsequently, we prepared PDA solutions at concentrations of 0.5, 2 and 5 mg/ml, each containing sufficient SDF-1. After the test, the final selected PDA concentration is 2 mg/ml. The microspheres were immersed in these solutions overnight, resulting in functionalized PMs. These PMs were then mixed with 5 mg/ml CuBG and stirred overnight, yielding PMs@CuBG. The microspheres were freeze-dried for characterization and further experimentation.

### Physical properties of the microspheres

A scanning electron microscope (SEM; FEISirion200, Philips, Amsterdam, Netherlands) was used to analyze the surface morphology and microstructure of randomly selected lyophilized PMs and PMs@CuBG microspheres. The pore size and particle size of the microspheres were determined using a nanoparticle size potentiometer (Malvern ZetasizerNanoZS90, Australia), and the data were tallied using Image J. The microspheres’ pore and particle sizes were determined using a SEM (FEISirion200, Philips, Amsterdam, The Netherlands).

### Drug release from the microspheres

#### Release of KGN

Ten milligrams of lyophilized PMs@CuBG microspheres was immersed in 10 ml of phosphate-buffered saline (PBS) solution at pH 7.4, and then positioned within a shaking incubator set to 37°C, oscillating at 70 rpm, for a period of 25 days. Sampling was conducted at predetermined time points: 0.5, 1, 3, 7, 14, 28, and 36 days. During each sampling, 2 ml of the supernatant was removed by centrifugation, with an equal volume of PBS added to replenish the solution. The harvested supernatants were stored at −20°C until the final measurements. Based on a pre-established standard curve, the release amount of KGN from the microspheres was determined at a wavelength of 287.4 nm using an ultraviolet spectrophotometer.

#### Release of SDF-1

The pre-prepared microspheres were immersed in three separate 50 ml centrifuge tubes containing 2 g of dichloromethane, centrifuged at 15 000 rpm for 5 min, the supernatant was removed, and the procedure was repeated three times to ensure that all the SDF-1 was precipitated. After resuspension with 2 ml PBS, the amount of SDF-1 carried by the microspheres was measured by ELISA kit. Similarly, the amount of SDF-1 released at each time point was detected, and the release ratio was calculated and the release curve was plotted.

#### Release of CuBG

Fifty milligrams of lyophilized PMs@CuBG microspheres was randomly selected and immersed in Tris-HCl buffer solution. The microspheres were then placed in a 37°C shaking incubator and agitated at 70 revolutions per minute (rpm) for 25 days. The Tris-HCl buffer solution was exchanged and collected at predetermined time points: 0.5, 1, 3, 7, 14, 28 and 42 days. The concentration of released Cu^2+^ in the solution was determined using Inductively Coupled Plasma Mass Spectrometry (ICP-MS; Agilent 5110, USA).

### Degradation of the microspheres

In the aforementioned drug release experiment for CuBG, a portion of the microspheres were collected at 14, 28 and 42 days. These microspheres were then freeze-dried and their morphology was observed using SEM.

### Biocompatibility of the microspheres

The cell viability was quantitatively analyzed using a live/dead cell assay kit (Invitrogen, Carlsbad, CA). Initially, BMSCs were seeded at a density of 1.0 × 10^4^ cells/ml in the lower chamber of a 24-well culture plate, while the PMs@CuBG microspheres were placed in the upper chamber at a concentration of 50 mg/ml for co-culture. After incubation periods of 1, 3 and 5 days, the culture medium was discarded, and the wells were thoroughly washed with PBS. A freshly prepared 250 µl calcein AM/PI staining solution was added to each well, followed by incubation in the dark at 37°C on a shaker for 30 min. The samples were then observed under a confocal fluorescence microscope (Zeiss Axiovert 200; Carl Zeiss).

Similarly, BMSCs were seeded in the lower chamber of a 96-well plate, and the microspheres were placed in the upper chamber for co-culture. After incubation periods of 1, 3 and 5 days, 10 μl of CCK-8 solution was added to the lower chamber. Following 1 h of incubation in the dark at 37°C on a shaker, the absorbance was measured at a wavelength of 450 nm using a multi-mode microplate reader.

### Impact of microspheres on cell migration

In the Transwell system (8 μm pores, Corning, USA), BMSCs were seeded in the upper chamber at a density of 3.0 × 10^4^ cells/ml, while the microspheres were placed in the lower chamber at a concentration of 10 mg/ml. Both chambers were filled with a culture medium containing 10% fetal bovine serum (FBS) and co-cultured for 24 h. After fixing the cells on the membrane, they were stained with a 0.5% crystal violet solution (Macklin, China). Non-migratory cells remaining in the upper chamber were gently removed with a cotton swab. The migrated cells were imaged and counted using a bright-field microscope for analysis.

A scratch wound healing assay was carried out utilizing a Transwell system (Corning, USA). Cells were sown in the lower compartment and allowed to grow until 70-80% confluence. A 200 μl pipette tip was used to draw a straight line across the center of the well’s bottom. After washing the dissociated cells with PBS, the microspheres were put in the top chamber. The cells were cultured in a serum-free medium. Scratch closure was observed, and images were taken with a bright-field microscope at 0, 12 and 24 h. Images were processed with ImageJ software to determine the level of wound healing.

### Effects of microspheres on macrophages

#### Immunofluorescence

The experiments were classified as blank, PMs, PMs@CuBG, LPS, LPS + PMs and LPS + PMs@CuBG. Macrophages were seeded in the lower chamber, while the microspheres were placed in the upper chamber at a concentration of 10 mg/ml. Both chambers were filled with a culture medium containing 10% FBS. After 7 days of co-culture with microspheres, the cells were rinsed with PBS and fixed with 4% paraformaldehyde on ice for 25 min. Permeabilization was carried out for 1 h with 0.3% Triton X-100 (Sigma-Aldrich, St Louis, MO, USA). Blocking was done with a specialized solution (Biosharp, Shanghai, China). Primary antibodies to CD206 and iNOS (Abcam, Cambridge, UK) were incubated overnight on a shaker at 4°C. After washing with PBS, the appropriate secondary antibodies (1:200; Servicebio, Ghent, Belgium) and phalloidin (1:1000, Yeasen, Shanghai, China) were added and incubated on a shaker at 37°C for 1 h. After washing with PBS, the cell nuclei were stained with DAPI (Abcam) for 10 min. Following washing, confocal microscopy observations (Zeiss Axiover200; Carl Zeiss) were performed to collect pictures. ImageJ was used to do a semi-quantitative examination of fluorescence.

#### Real-time quantitative reverse transcription polymerase chain reaction

Cells were washed with PBS after being cultured for up to 7 days using the experimental grouping outlined above. Total RNA was extracted with Trizol reagent, and its concentration and purity were measured using absorbance at 260 and 280 nm. cDNA was produced from 1 μg of RNA using the Revert Assistant First-strand cDNA Synthesis Kit. The SYBR PreMix Ex Taq Kit and ABI 7500 Sequence Detection System were then used to perform real-time quantitative reverse transcription polymerase chain reaction (qRT-PCR) analysis. Gene expression levels of VEGF (Vascular Endothelial Growth Factor), TNF (Tumor Necrosis Factor)-α, PDGF (Platelet-Derived Growth Factor), and TNF-β were measured using primers provided in Supplementary Figure S2 for standardization. The results were examined using the comparative CT method (ΔΔCT method), and all tests were repeated three times.

### Influence of microspheres on chondrocytes

#### Alcian blue staining

Transwell system was used to co-culture microspheres and SMSCs in 24-well plates. Microspheres were inoculated in the upper compartment and cells in the lower compartment. Both chambers were filled with culture medium containing 10% FBS. After 2 weeks, the culture media was withdrawn and the cells rinsed with PBS. The cells were then fixed with 4% paraformaldehyde on ice and rinsed again with PBS. Cells were then stained with 1% Alcian Blue solution for 30 min at room temperature. An upright light microscope was used to observe and acquire images for analysis.

#### Immunofluorescence

Rat macrophages were treated with LPS overnight and inoculated in the upper chamber. The microspheres were placed in the lower chamber at a concentration of 10 mg/ml to the SMSCs suspension before initiating the co-culture. The experimental groups consisted of blank, PMs, and PMs@CuBG. After 3 weeks of co-culturing, col-2α fluorescent intensity was detected using immunofluorescence.

### Therapeutic effects of microspheres *in vivo*

#### Establishment of the OA model in rat knee joints

The construction of the OA rat model was approved by the Ethics Committee of the Affiliated Suzhou Hospital of Nanjing Medical University. The approval number for animal experimentation is K-2024-058. All procedures were carried out in accordance with the requirements of the National Institutes of Health (USA). The experiment used male Sprague–Dawley rats, averaging 12 weeks old, provided by the Experimental Animal Center of Soochow University, totaling 30 rats. The rats were divided into four experimental groups, with seven rats in each group, and two additional rats were kept as backups. The experiment employed the method of medial meniscectomy. Rats were anesthetized with an intraperitoneal injection of 2% sodium pentobarbital at a dosage of 2.5 ml/kg. After hair removal and disinfection, sterile instruments were used to longitudinally incise the skin from the distal end of the femur to the proximal end of the tibial plateau, exposing the right knee joint. The collateral ligament was transected horizontally, and the joint cavity was opened. The medial meniscus was then excised. Following this, the joint cavity was closed, and after disinfection, the layers were sutured sequentially. In the Sham group, only the joint cavity was opened without resection of the meniscus or damage to the ligaments. Adequate food, water, and other resources were provided post-surgery. Post-surgery, rats underwent running on a custom-made rat treadmill three times a week at a speed of 20 m/min for 1 month to induce OA in the knee joints. Four weeks post-surgery, rats in the surgical group were divided into PBS group, PMs group and PMs@CuBG group, and corresponding drugs were injected into the joint cavities of rats in each group. The concentration is 10 mg/ml and the dose is 100 μl. After 4 weeks of unrestricted activity, they were euthanized for subsequent experiments.

#### Morphological analysis of the rat knee joint

After euthanasia, the right knee joints were excised. Tissues were cut layer by layer to expose the knee joint. The anterior and posterior cruciate ligaments were severed to fully expose the tibial plateau. The specimens were then observed under a stereomicroscope.

#### Radiographic analysis of the rat knee joint

Following euthanasia, the right knee joints were excised and imaged using Micro-CT (SkyScan 1176; Aartselaar, Belgium). The volume of osteophytes in six rats per group was calculated using CTAn software (Blue Scientific, Cambridge, UK).

#### Rat joint histological analysis

The right knee joints of the rats were harvested and fixed in 4% paraformaldehyde for 1 day, followed by decalcification in 10% ethylenediaminetetraacetic acid (EDTA) for 1 month. The tissues were then embedded in paraffin, sectioned at 5-μm thickness, stained, and the results were analyzed using ImageJ software.

#### Picro-Sirius red and fast green staining

Joint tissue sections were deparaffinized and hydrated, then rinsed under running water for 3 min. The sections were sequentially immersed in Picro-Sirius red and fast green solutions. After 1 min, they were rinsed under running water for another 3 min. The sections were then dipped three times in a container filled with acid alcohol, rinsed under tap water, and observed under a microscope.

#### Immunohistochemical staining

Tissue sections were deparaffinized and hydrated, then rinsed with tap water. They were digested with 5 μg/ml hyaluronidase at 37°C for 30 min. Antigen retrieval was performed with 6% H_2_O_2_, followed by incubation for 20 min. After washing, sections were blocked with 10% rabbit serum at room temperature for 1 h. The col-2α antibody was diluted 1:200 in BSA-PBS solution and incubated overnight at 4°C. Following further washing, the secondary antibody was diluted 1:600 in BSA-PBS solution and applied to the sections, which were then left undisturbed for 1 h. After additional washes, sections were incubated with biotinylated-horseradish peroxidase reagent in the dark for 0.5 h. Color development was achieved by applying the chromogen solution to the sections and observing under a microscope for DAB color development. The reaction was stopped based on the degree of color development. Finally, sections were counterstained with hematoxylin to stain cell nuclei, mounted, and observed under a microscope.

### Data analysis

Data analysis for this study was conducted using GraphPad Prism 8.0 software. Experimental results are presented as mean ± standard deviation. One-way analysis of variance and independent samples *t*-tests were utilized for statistical comparisons (ns = no significant difference, *P < 0.05, **P < 0.01, ***P < 0.001, ^#^*P* < 0.05, ^##^*P* < 0.01, ^###^*P* < 0.001).

## Results and discussion

### Characterization of microspheres

**Figure 2.**
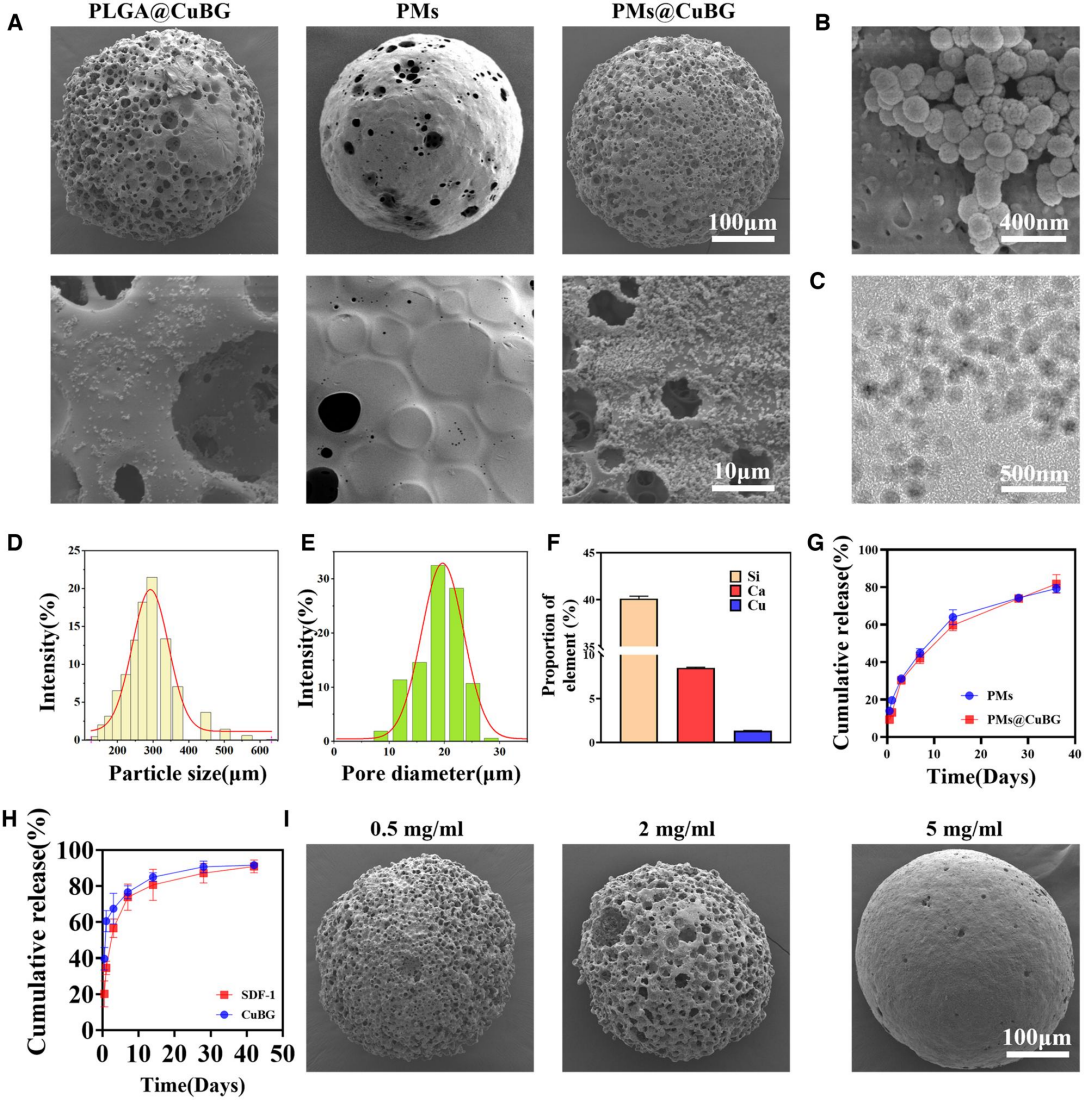
Characterization of microspheres. (**A**) SEM images of PLGA@CuBG, PMs and PMs@CuBG. (**B**) Surface SEM images of PMs@CuBG in **A**. (**C**) TEM images of CuBG. (**D**) Particle size analysis of PMs@CuBG. (**E**) Pore size analysis of PMs@CuBG. (**F**) Elemental analysis of PMs and PMs@CuBG. (**G**) Release curves of KGN releasing from PMs and PMs@CuBG (*n* = 3). (**H**) Release curves of SDF-1 and CuBG releasing from PMs@CuBG (*n* = 3). (**I**) SEM images of PMs@CuBG coatings with different PDA concentration.

PLGA@CuBG, PMs and PMs@CuBG were observed to confirm that the surface of PMs is coated with a thin film of PDA compared with PLGA microspheres without PDA. PMs@CuBG had greater CuBG adhesion than PLGA@CuBG. Observing CuBG on the surface of PMs@CuBG via SEM at 50 000× magnification revealed that the BG was clustered on the surface of the microspheres, possibly caused by localized hydrogen bonding (Figure 2A and B). In the solution, CuBG was evenly disseminated with an average size of approximately 118 ± 12.1 nm (Figure 2C). Elemental analysis using inductively coupled plasma optical emission spectrometry revealed that CuBG’s elemental content ratio was Si:Ca:Cu = 40.11%:8.49%:1.36% (Figure 2F). This result demonstrated that the BG is uniformly doped with copper. The average particle size in PMs@CuBG was 287.2 ± 15.1 µm with a pore size of 19.02 ± 4.25 µm. These microspheres closely matched those from our previous results, and the microsphere homogeneity was good (Figure 2D and E). Cells are reported to be able to pass through pores larger than 15 µm. Thus, the microsphere and pore sizes were compatible with our purpose. Elemental mapping of PMs@CuBG microspheres in Figure 2A revealed that Si elements were dispersed across the surface of the microspheres, indicating that the surface of PMs@CuBG microspheres had a substantial amount of CuBG (Supplementary Figure S3). Based on the supernatant measurements at various time intervals, it was discovered that 13.13% of KGN in PMs@CuBG was released within 24 h, and 80% of KGN was released gradually over the next 36 days (Figure 2G). The release of less than 90% of the total amount may be caused by loss during overnight stirring in CuBG and PDA solution and by incomplete degradation of the microspheres *in vitro* after 36 days. Within 24 h, 34.53% of SDF-1 and 60.53% of CuBG were released (Figure 2H), showing that both were rapidly released by the detachment of the PDA film.

Next, we explored various concentrations of PDA solution to improve the thin coating on the surface of the microspheres. A 0.5 mg/ml PDA solution resulted in a thin coating covering only the pores, possibly due to the force generated during stirring. In contrast, the film created by a 5 mg/ml PDA solution almost fully encased the microspheres, potentially resulting in no openings on the surface and preventing cells from entering the microspheres (Figure 2I). The film generated by a 2 mg/ml PDA solution, which moderately enclosed the surface of microspheres and broke down after 3 days, left the pores sufficiently exposed as to not impair the entry of cells (Supplementary Figure S4). According to the experimental results, the concentration of PDA selected for the PMs prepared by subsequent relevant experiments was 2 mg/ml.

**Figure 3.**
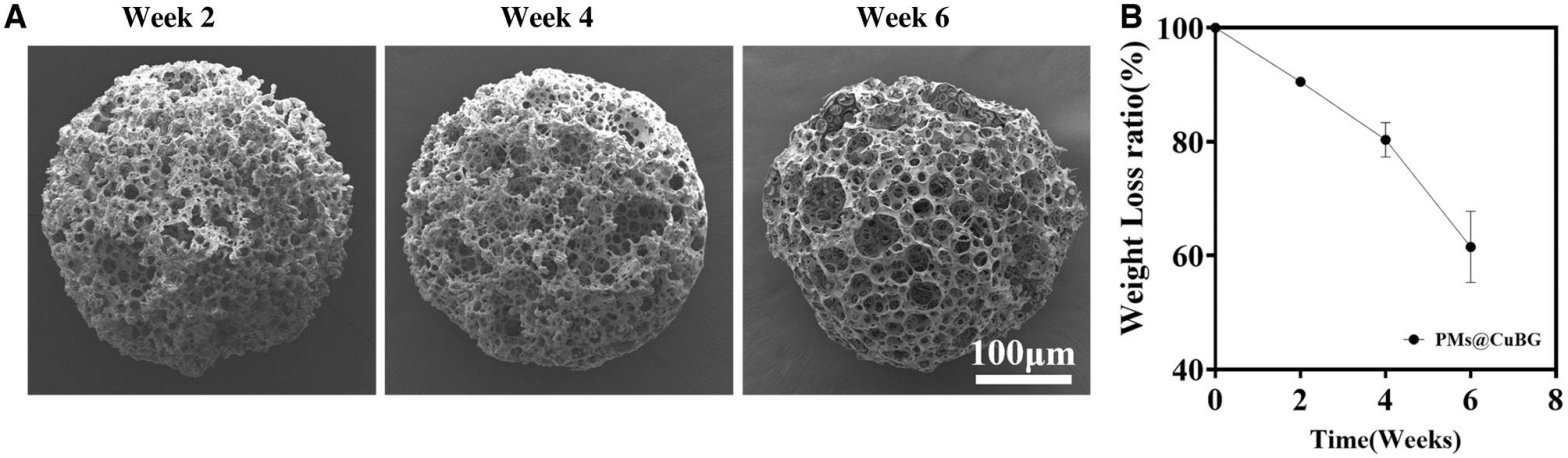
Degradation of the microspheres. (**A**) SEM images of PMs@CuBG degradation. (**B**) Residual mass change of PMs@CuBG (*n* = 3).

Finally, the degradation of microspheres was examined. To replicate microsphere breakdown *in vivo*, microspheres were immersed in PBS and shaken at 37°C. After 2, 4 and 6 weeks of immersion, a sample of microspheres was extracted and lyophilized to view by SEM. At 2 weeks, the PDA layer was entirely gone, the internal pores were exposed, and the microspheres were decreased in size while maintaining the majority of their original architecture (Figure 3A). After 4 weeks of degradation, the microspheres revealed structural damage, and after 6 weeks, they had degraded to 62% of their initial size (Figure 3B).

### Biological function of microspheres

**Figure 4.**
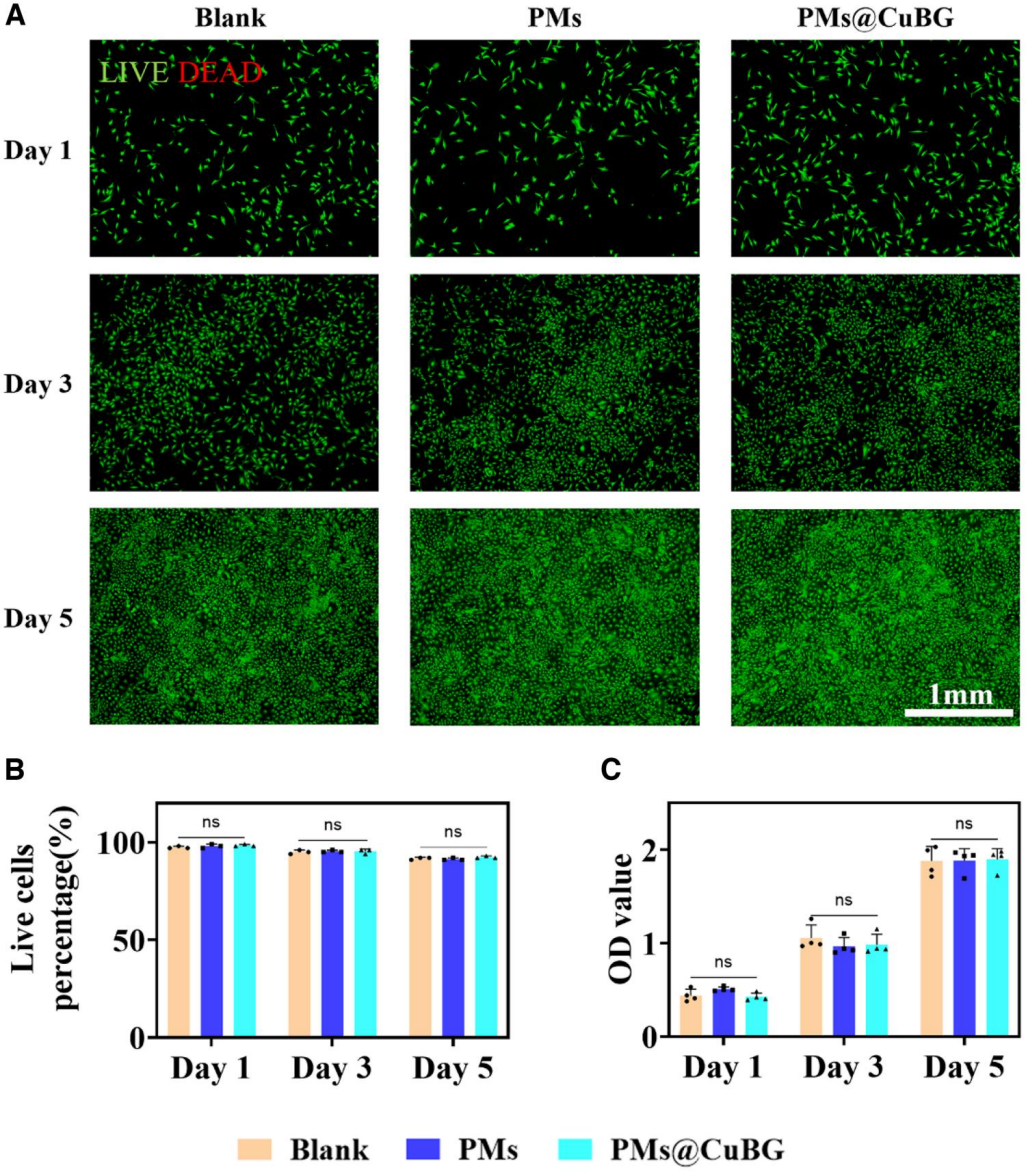
Biocompatibility of microspheres. (**A**) Live (green)/dead (red) fluorescence results of blank, PMs, PMs@CuBG groups on 1, 3 and 5 days. (**B**) CCK-8 assay of SMSCs (*n* = 3). (**C**) Live cells percentage obtained from the live/dead staining assay (*n* = 4) (ns: non-significant).

Next, we looked into the bioactivity of the microspheres. Synovial MSCs were co-cultured with PMs@CuBG, and their biocompatibility was determined using a live/dead assay and the CCK-8 assay. The live/dead assay revealed that cell survival was high during co-culture, with cells in the PMs@CuBG group maintaining a 92% survival rate by day 5 (Figure 4A and B). The CCK-8 assay gave similar results (Figure 4C). After adding PMs or PMs@CuBG to the co-culture, there was no statistically significant difference in synovial MSC bioactivity compared with the control group. This shows that the microspheres are biocompatible and can support cell colonization.

**Figure 5.**
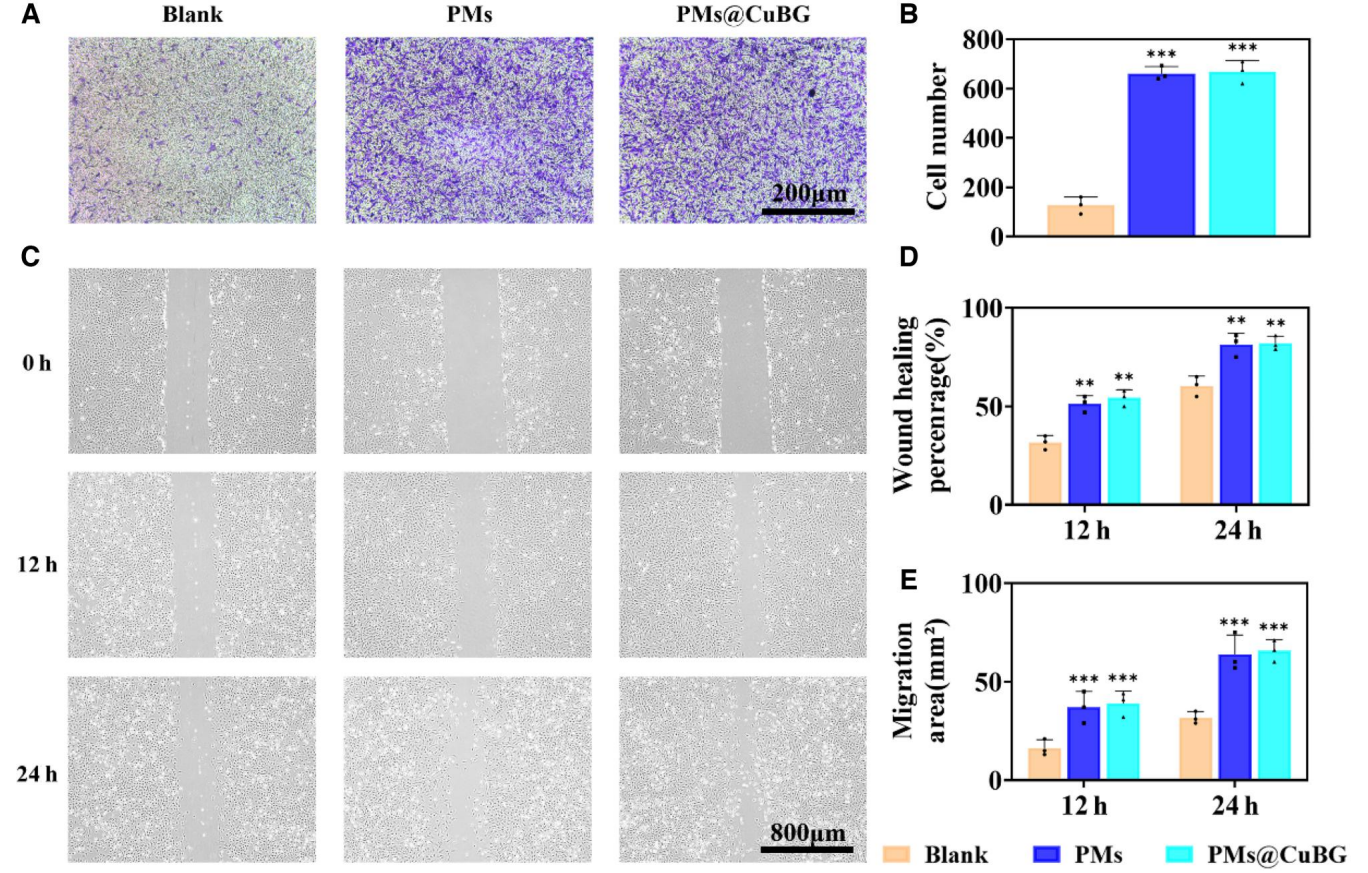
Impact of microspheres on cell migration. (**A**) Representative images of optical microscope showing cells migrated from upper chambers. (**B**) The number of migrated cells after 24 h culture. (*n* = 3). (**C**) Representative optical microscopic images of scratch line at 0, 12 and 24 h. The wound healing percentage analysis (**D**) and migration area analysis (**E**) of blank, PMs and PMs@CuBG group (*n* = 3) (**P < 0.01, ***P < 0.001, when compared with the blank group).

We used transwell migration and scratch assays to test the microspheres' potential to recruit cells. We discovered that SDF-1-loaded microspheres caused more cells to cross the transwell membrane (Figure 5A and B). In the scratch assay, cell migratory ability followed a similar pattern with greater migration by cells co-cultured with PMs and PMs@CuBG (Figure 5C). The wound healing percentage of synovial MSCs in the PMs@CuBG group was 54.3% and 82% after 12 and 24 h, respectively, with migration areas of 39 and 65.7 mm^2^ (Figure 5D and E). The PMs group showed similar results. Based on our previous research, the application of E7 (a stem cell mobilization drug) within the joint has confirmed the recruitment of CD73 (+) CD90 (+) cells within the joint space, which are considered to be MSCs [19, 24]. Current studies have shown that the intra-articular administration of SDF-1 can enhance the recruitment of endogenous MSCs [23]. Therefore, we hypothesize that the addition of SDF-1 in the microspheres could facilitate the recruitment of MSCs within the knee joint. Based on the current evidence, we speculate that PMs@CuBG can recruit synovial MSCs to the joint. To get to the bottom of this, we injected PMs and PMs@CuBG into the joint cavity of rats, and after 7 days of retention, we killed the rats and removed the microspheres in the joint cavity for immunofluorescence experiments, and CD73(+) CD90(+) cells could be clearly observed, indicating that PMs and PMs@CuBG had the ability to recruit synovial MSCs into the joint cavity (Supplementary Figure S6).

### Effect of microspheres on macrophages

**Figure 6.**
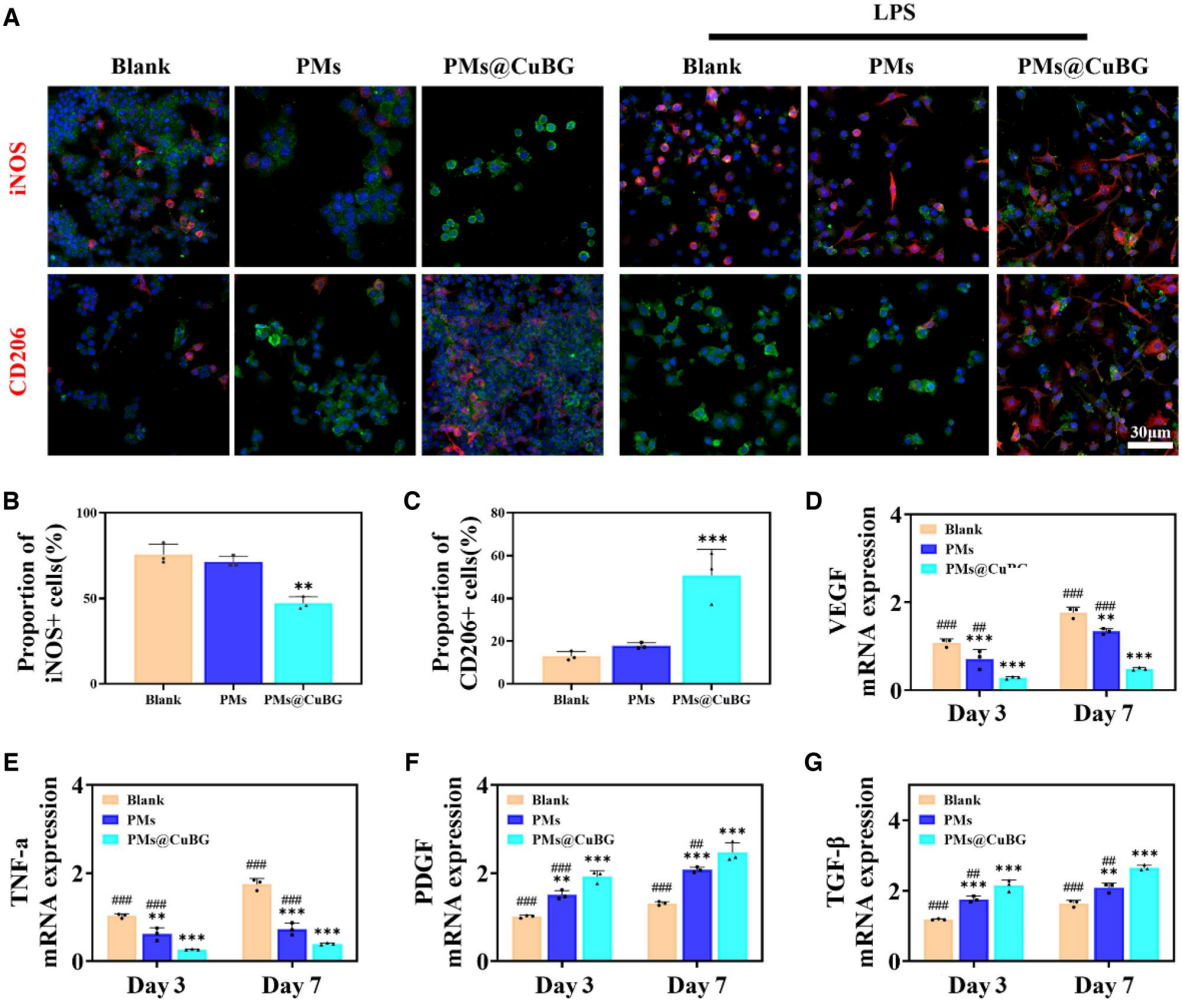
Effect of microspheres on macrophages. (**A**) Immunofluorescence staining of iNOS and CD206. Blue: DAPI, green: F4/80. Statistics of iNOS (**B**) and CD206 (**C**) positivity rates (*n* = 3). The mRNA expression analysis of VEGF (**D**), TNF-α (**E**), PDGF (**F**) and TGF-β (**G**) in the blank, PMs and PMs@CuBG group and PMs@met group on the third day and the seventh day (*n* = 3) (**P < 0.01, ***P < 0.001, when compared with the blank group. ^##^*P* < 0.01, ^###^*P* < 0.001, when compared with the PMs@CuBG group).

Macrophages are widely dispersed in synovial tissue and have a significant impact on the progression of OA [25]. During OA, cartilage debris, aggregated proteins, fibronectin, and intracellular proteins within necrotic cells act as danger-associated molecular patterns that stimulate the polarization of M0 macrophages into M1 macrophages, which produce inflammatory cytokines and chemokines, and induce an intra-articular inflammatory milieu that further exacerbates cartilage degradation while negatively affecting the efficacy of regenerative therapies with *in situ* MSCs [26]. Changing the phenotype of synovial macrophages from M1 to M2 may improve the efficacy of cell therapy. Therefore, we looked into the role of microspheres in macrophage polarization. Microspheres and macrophages were co-cultured by Transwell system. On day 7 of culture, immunofluorescence labeling was conducted, and macrophages were observed using confocal microscopy (Figure 6A). We found that in cells that did not receive lipopolysaccharide (LPS) treatment, CD206 expression increased following treatment with PMs@CuBG, indicating that the fraction of M2 macrophages rose dramatically. This trend was not evident in the PMs group. After LPS treatment, the proportion of M1 pro-inflammatory phenotype marker iNOS (+) cells was significantly reduced to 47%, compared to 75% in the blank group and 71% in the PMs group, while the proportion of cells positive for the M2 anti-inflammatory phenotype marker CD206 was significantly higher in the PMs@CuBG group compared with other groups (Figure 6B and C). These results suggest that PMs@CuBG might polarize M0 and M1 macrophages to M2 macrophages. We believe this is caused by CuBG, as the presence of Cu^2+^ promotes polarization toward M2 macrophages. To confirm the effect of PMs@CuBG on macrophages, we performed RT-qPCR on days 3 and 7. PMs@CuBG significantly reduced VEGF and TNF-α expression while increasing PDGF and TGF-β expression relative to the control group (Figure 6D–G). This supports our conclusion that macrophages were polarized toward the M2 phenotype by PMs@CuBG treatment.

### Promoting cartilage differentiation function of microspheres

**Figure 7.**
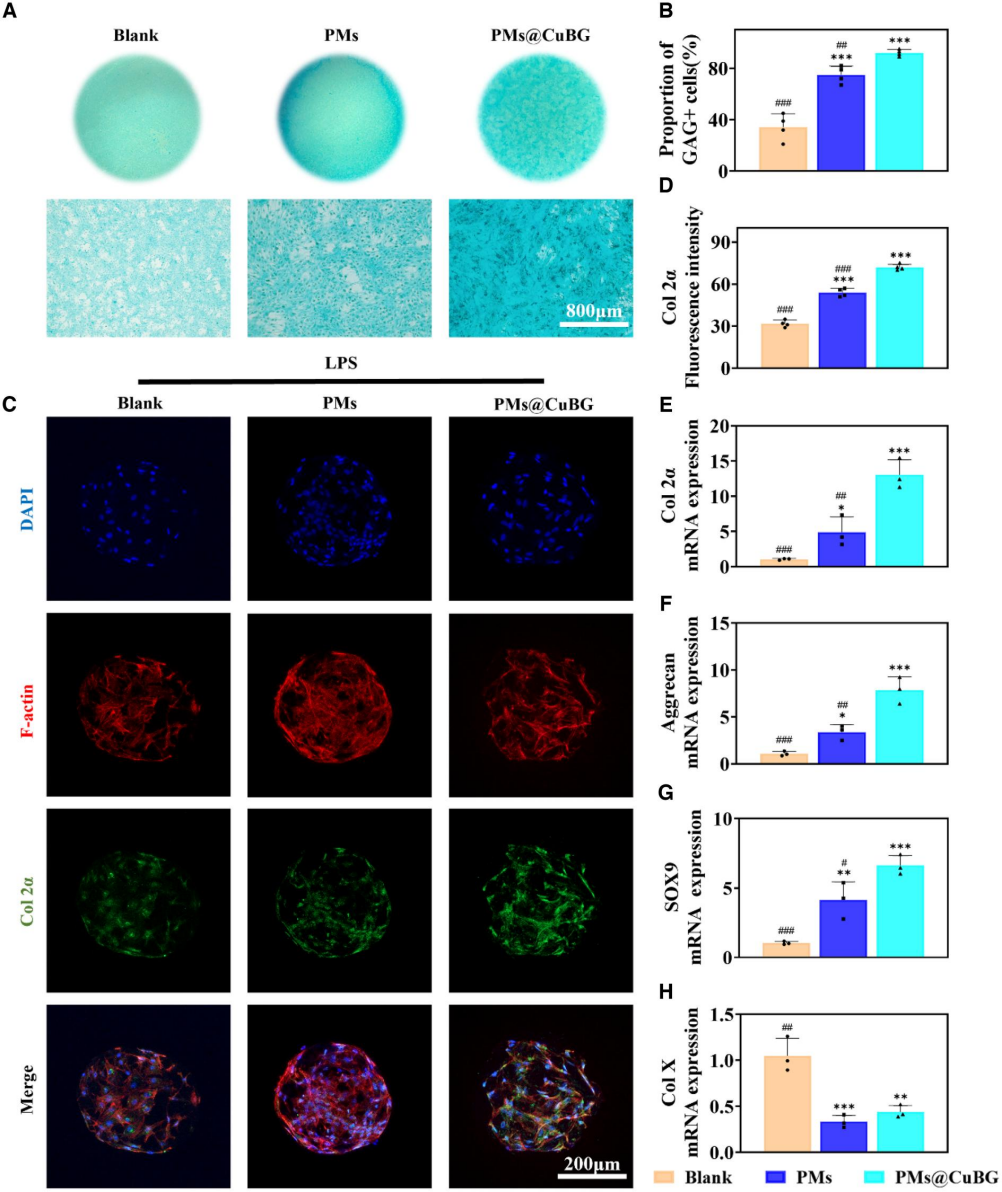
Promoting cartilage differentiation function of microspheres. (**A**) Representative images of Alcian blue staining after 2 weeks of chondrogenic culture. (**B**) Statistics of GAG+ positivity rates (*n* = 3). (**C**) Quantitative analysis of immunofluorescence intensity of col 2α (*n* = 3). (**D**) Immunofluorescence staining of col 2α expression from SMSCs-loaded microspheres on the 21st day. (**E**–**H**) The relative mRNA expression of chondrogenic genes (col 2α, aggrecan and SOX9), and hypertrophic gene collagen X after 21 days co-culture (*n* = 3) (*P < 0.05, **P < 0.01, ***P < 0.001 when compared with the blank group. ^#^*P* < 0.05, ^##^*P* < 0.01, ^###^*P* < 0.001 when compared with the PMs@CuBG group).

The extracellular matrix (ECM) of chondrocytes is a critical component of cartilage tissue which contains collagen, proteoglycans, and water [27, 28]. Col-2α is the primary collagen found in the cartilage ECM, while glycosaminoglycans (GAGs) are key components of proteoglycans [29]. We detected these two indicators to assess the chondrogenic differentiation ability of microspheres. A transwell system was used to co-culture microspheres and SMSCs in 24-well plates. Microspheres were inoculated in the upper compartment and cells in the lower compartment. After co-culturing SMSCs with microspheres, Alcian blue staining was used to assess GAG expression levels. The level of staining in the PMs and PMs@CuBG groups was considerably higher than in the blank group, demonstrating that these two types of microspheres might enhance GAG secretion via the delayed release of internally loaded KGN. It was also discovered that the expression of GAGs was higher in the PMs@CuBG group than in the PMs group, leading us to believe that CuBG has a favorable function in chondrogenic expression (Figure 7A and B). To validate the pro-chondrogenic gene expression activity of microspheres, rat macrophages were challenged with LPS overnight, and a co-culture system with synovial MSCs was constructed using transwell chambers to assess the chondrogenic ability of microspheres in an inflammatory setting. After LPS intervention, macrophages were inoculated in the upper compartment and microspheres and synovial MSCs were inoculated in the lower compartment. After 21 days of co-culture, the cells were examined by immunofluorescence (Figure 7C). The PMs@CuBG group exhibited higher col-2α expression compared with other groups (Figure 7D). This could be because CuBG polarized macrophages from M1 to M2, altering the inflammatory milieu in the system and allowing synovial MSC development and chondrogenic gene expression. We used RT-qPCR to determine the expression of typical chondrocyte marker genes such as col-2α, AGG, SOX9, and col-X. PMs@CuBG enhanced the expression of chondrogenesis-related genes including col-2α, AGG, and SOX9. The col-X results showed that PMs@CuBG did not promote cells to become hypertrophic chondrocytes (Figure 7E–H). Based on these findings, we confirmed that PMs@CuBG could alter the inflammatory environment by reprogramming macrophages, improve the chondrogenic differentiation of MSCs cells, and promote the generation of ECM, potentially leading to cartilage repair.

### *In vivo* therapeutic effects of microspheres

**Figure 8.**
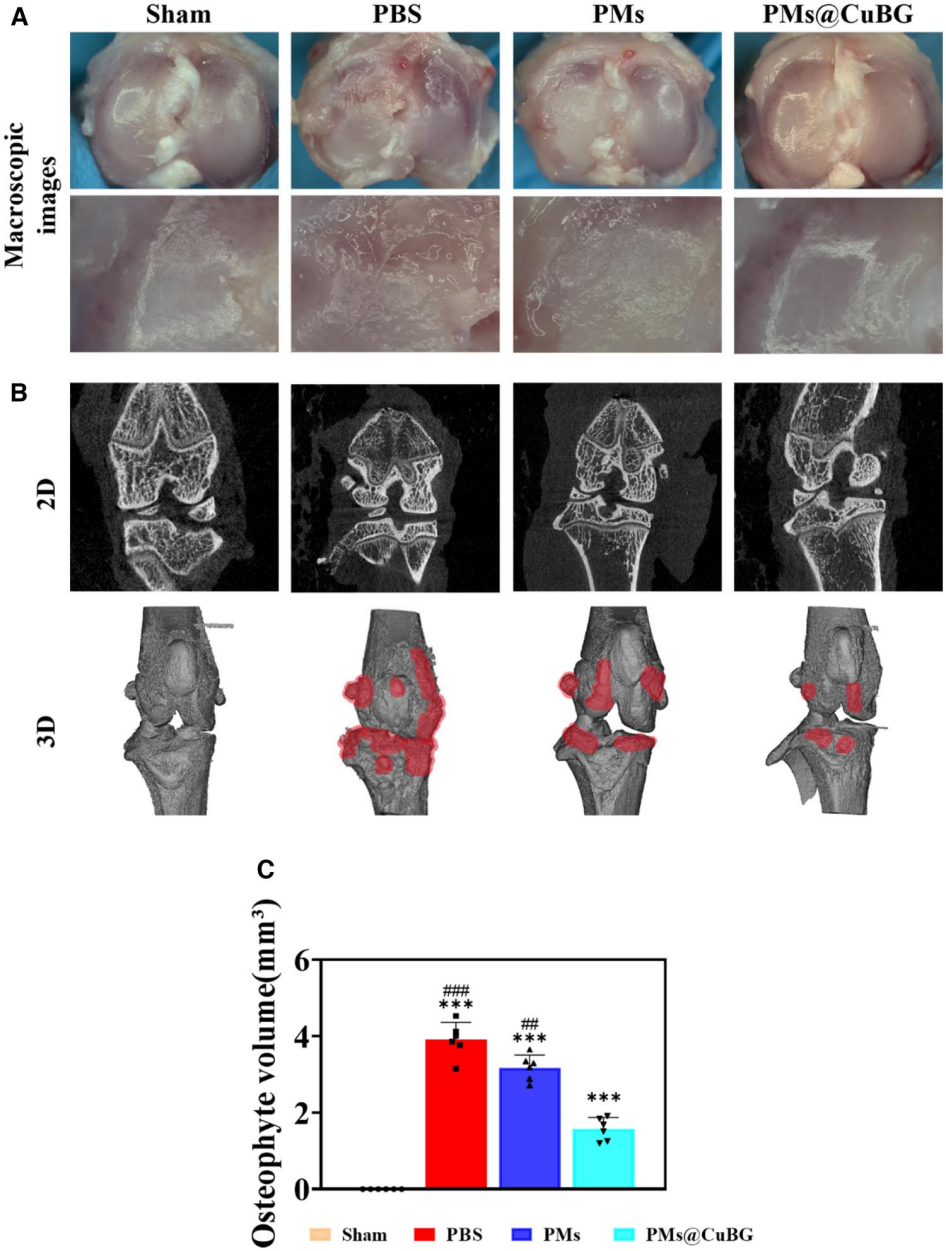
The performance of PMs@CuBG treat osteoarthritis model *in vivo*. (**A**) Macroscopic observations. (**B**) 2D and 3D representative CT images of the rat’s knee joint at 6 weeks postoperatively. (**C**) The relative volume of total osteophytes at 6 weeks (*n* = 6) (***P < 0.001 when compared with the sham group. ^##^*P* < 0.01, ^###^*P* < 0.001 when compared with the PMs@CuBG).

To further evaluate the efficacy of PMs@CuBG *in vivo*, we created a rat knee OA model using medial meniscectomy and exercise intervention as performed in previous studies [19]. Following a 1-month exercise intervention, rats were divided into groups and injected with PBS, PMs, or PMs@CuBG microspheres into the knee joint every 2 weeks until being sacrificed after 6 weeks to isolate the knee. The injection concentration of microspheres was 10 mg/ml and the injection dose was 100 μl. The knee joints were examined using stereo microscope and micro-CT. The appearance of the tibial plateau of the knee joints of OA rats could be seen by in-body microscopy. The tibial surfaces of the knee joints of the rats in the sham group were smooth with no obvious wear, while the synovial membrane at the articular surfaces of the rats injected with PBS and PMs after surgery showed clear wear, and the surfaces of the tibial plateau showed varying degrees of cartilage degeneration. In contrast, PMs@CuBG injection resulted in the mildest cartilage degeneration in the knee joint, comparable with the sham group (Figure 8A). At the same time, we used micro-CT to coronally scan and rebuild 3D images of the knee joint and marked osteophytes in the reconstructed images with red shadows, revealing osteoid development in the joint. We discovered that there was no osteophyte generation in the sham group, and the remaining groups had varying degrees of osteophyte generation, with the PMs@CuBG group having the fewest osteophytes. After quantifying the volume of osteophytes using CTAn, the statistical results were consistent with the above observations by in-body microscopy (Figure 8B and C).

**Figure 9.**
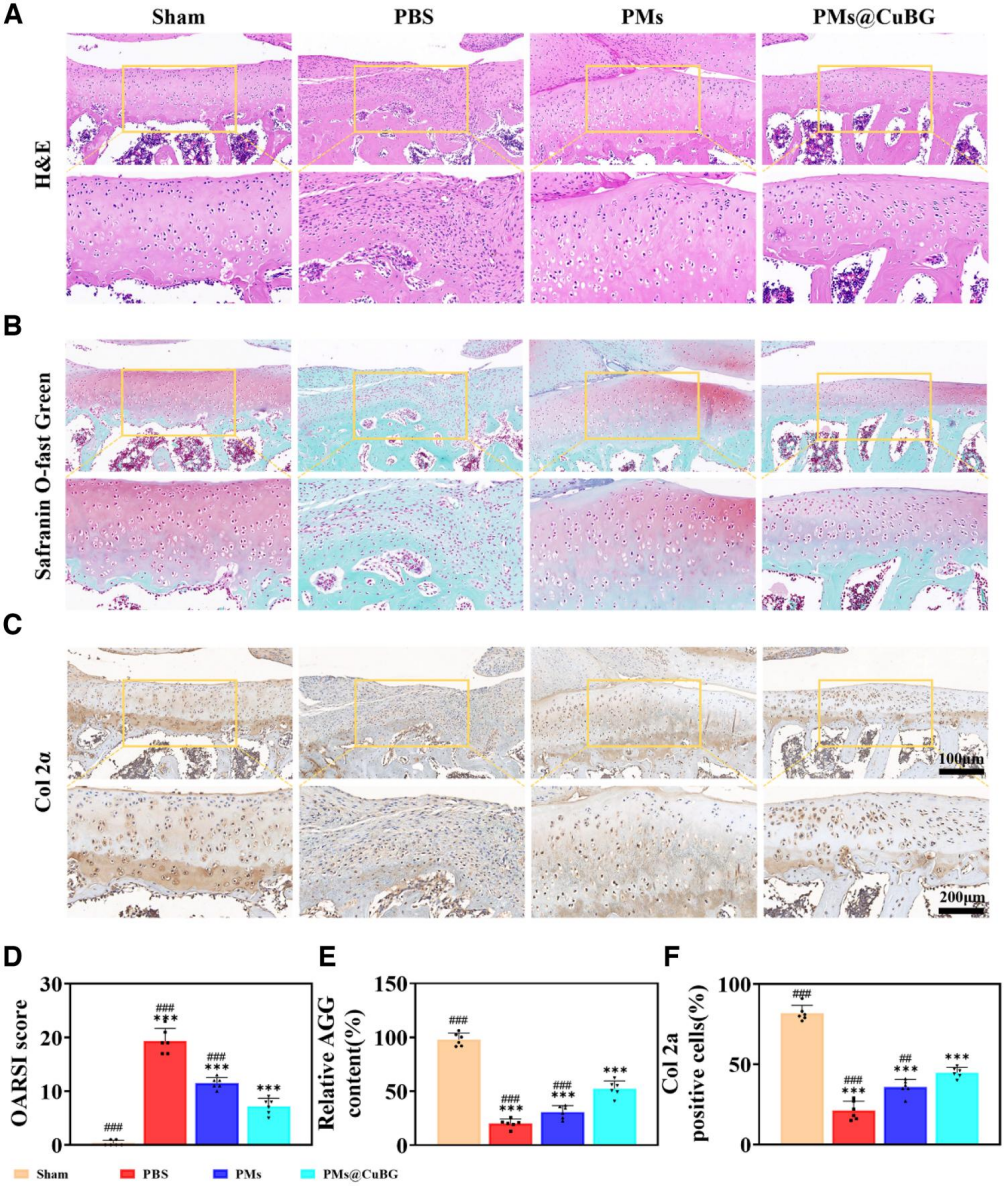
The performance of PMs@CuBG treat osteoarthritis model *in vivo*. (**A**) H&E stained image of the section. (**B**) Safranin O-fast green stained image of the section. (**C**) Col 2α immunohistochemical staining image of the section. (**D**) OARSI score (*n* = 6). (**E**) Relative content of aggrecan (*n* = 6). (**F**) Quantification of col 2α-positive cells (*n* = 6) (***P < 0.001 when compared with the sham group. ^##^*P* < 0.01, ^###^*P* < 0.001 when compared with the PMs@CuBG group).

Additionally, the isolated rat knee joints were fixed, decalcified, embedded, sectioned, and stained with hematoxylin-eosin (H&E) and safranin O-solid green. The tissue sections of tibial plateau were observed after staining. Microscopy revealed that the sham group's cartilage surface was flat with no evident flaws, the ECM was intact, and the chondrocytes were grouped in an ordered manner, which was consistent with previous findings. The cartilage in the PBS, PMs and PMs@CuBG groups displayed varied degrees of integrity deficiencies, ECM loss, and disorderly cell organization, all of which are observed in OA [30]. However, it is worth noting that the integrity loss was less severe in the PMs@CuBG group and closer to the sham group (Figure 9A and B). Col-2α immunohistochemical staining revealed that the PMs@CuBG group had a significantly higher proportion of positive cells compared with the PBS and PMs groups (Figure 9C). Using three distinct staining procedures, we calculated the Osteoarthritis Research Society International (OARSI) score with the recommended formula: score=grade×stage [31], aggrecan concentration and proportion of col-2α-positive cells. Except for the sham group, the PMs@CuBG group showed the lowest OARSI score, the highest aggrecan content, and the largest percentage of positive cells (Figure 9D–F). Immunohistochemical staining for CD206 and iNOS revealed a considerably larger percentage of macrophage M2 polarization in the PMs@CuBG group compared to the PBS and PMs groups (Supplementary Figure S7). All of these results indicate improved outcomes in the rat OA model following treatment with PMs@CuBG microspheres. It is worth noting that the PMs group displayed a therapeutic trend, which could be attributed to PDA’s ROS-scavenging function and KGN’s pro-chondrogenic differentiation effect. These findings reveal PMs@CuBG microspheres’ potential to repair cartilage injury in OA rats.

## Conclusion

Using microfluidic technology, a novel injectable microsphere, PMs@CuBG, was created by developing and integrating PLGA, PDA, CuBG, SDF-1 and KGN. The PMs@CuBG microspheres were able to ameliorate the inflammatory microenvironment by reprogramming synovial macrophages and promoting their polarization toward the anti-inflammatory M2 phenotype in the articular cavity. They were also able to recruit synovial MSCs *in situ* and promote their chondrogenic differentiation, thereby promoting the repair of damaged cartilage. These PMs@CuBG microspheres may prove to be a novel and effective strategy for the treatment of OA. At the same time, the downstream signaling mechanisms by which they function require further investigation.

## Supplementary Material

rbae142_Supplementary_Data

## Data Availability

The original contributions presented in the study are included in the article/Supplementary data. Further inquiries can be directed to the corresponding author.
