# Supplementary material for: Injectable microspheres filled with copper-containing bioactive glass improve articular cartilage healing by regulating inflammation and recruiting stem cells
Source: Regen Biomater. 2024 Dec 17;12:rbae142. doi: 10.1093/rb/rbae142 (PMC11751692; doi:10.1093/rb/rbae142)
Supplement: rbae142_Supplementary_Data [file rbae142_supplementary_data.docx]

**Supplementary information**

**Injectable microspheres filled with copper-containing bioactive glass improve articular cartilage healing by regulating inflammation and recruiting stem cells.**

Hua Gao ^a, †^, Eryu Ning ^a, †^, Xiaoyu Zhang ^a, †^, Zhiqiang Shao ^a^, Dan Hu ^a^, Lang Bai ^a,*^, Hui Che ^a,*^, Yuefeng Hao ^a,*^

^a^Orthopedics and Sports Medicine Center, The Affiliated Suzhou Hospital of Nanjing Medical University, Suzhou Municipal Hospital, Gusu School, Nanjing Medical University, 242 Guangji Road, Suzhou 215008, P. R. China.

^†^These authors contributed equally to this work.

*Corresponding authors:

Yuefeng Hao: 13913109339@163.com;

Hui Che: che_hui@aliyun.com;

Lang Bai: bailang887@163.com

**Figure S1: Composition of bioactive glass**

| **Element** | **SiO2** | **CaO** | **CuO** |
| --- | --- | --- | --- |
| **Weight percent (%)** | 86 | 12 | 2 |

**Figure S2: Primers used in qRT-PCR experiment**

| Primer name | Orientation | Sequence (5‘-3’) |
| --- | --- | --- |
| VEGF | Forward | GTCCGATTGAGACCCTGGTG |
|  | Reverse | CGGGATTTCTTGCGCTTTCG |
| TNF-α | Forward | TACTGAACTTCGGGGTGATTGGTCC |
|  | Reverse | CAGCCTTGTCCCTTGAAGAGAACC |
| PDGF | Forward | CGCTCTTCCTTCCTCTCTGC |
|  | Reverse | AGTGGTCGCTCAGCATTTCA |
| TGF-β | Forward | CTTCAGCTCCACAGAGAAGAACTGC |
|  | Reverse | CACGATCATGTTGGACAACTGCTCC |
| COL-II | Forward | CGCCACGGTCCTACAATGTC |
|  | Reverse | TGCACCCCTCTCTCCCTTGT |
| SOX9 | Forward | TACGACTACACCGACCACCA |
|  | Reverse | TTAGGATCATCTCGGCCATC |
| Aggrecan | Forward | TGGCATTGAGGACAGCGAAG |
|  | Reverse | TCCAGTGTGTAGCGTGTGGAAATAG |
| COL-X | Forward | AAGTGGACCGAAAGGAGACA |
|  | Reverse | TGGAAACCCATTCTCACCTC |
| GAPDH | Forward | AGTGCCAGCCTCGTCTCATA |
|  | Reverse | GGTAACCAGGCGTCCGATAC |


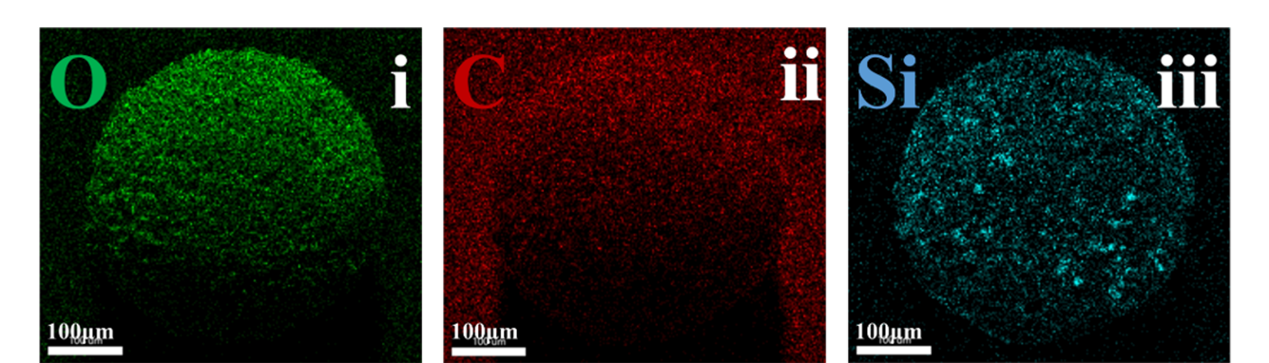


**Figure S3: Elemental mapping images of PMs@CuBG in Figure 1: i) oxygen (O) map, ii) carbon (C) map, and iii) silicon** **(Si) map.**


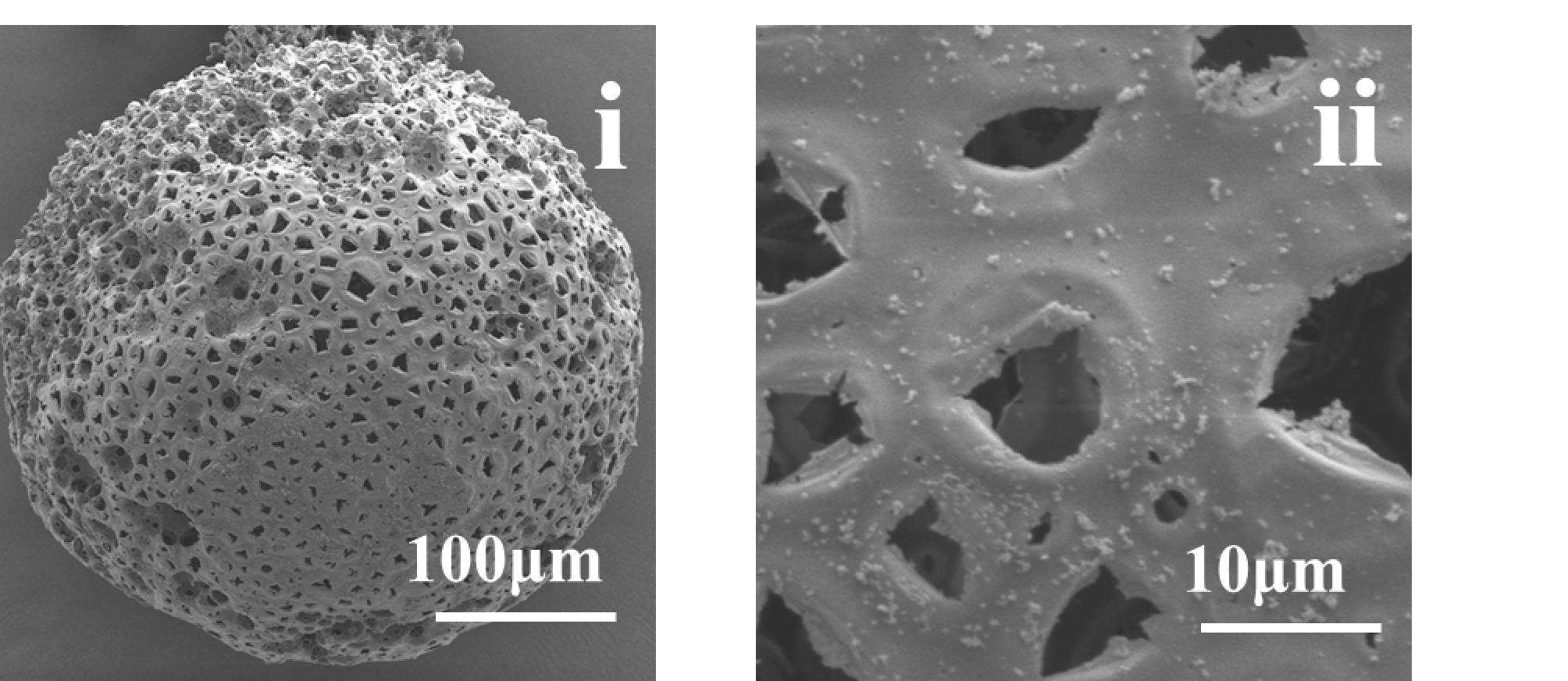


**Figure S4: SEM images of PMs@CuBG degradation after 3 days.**


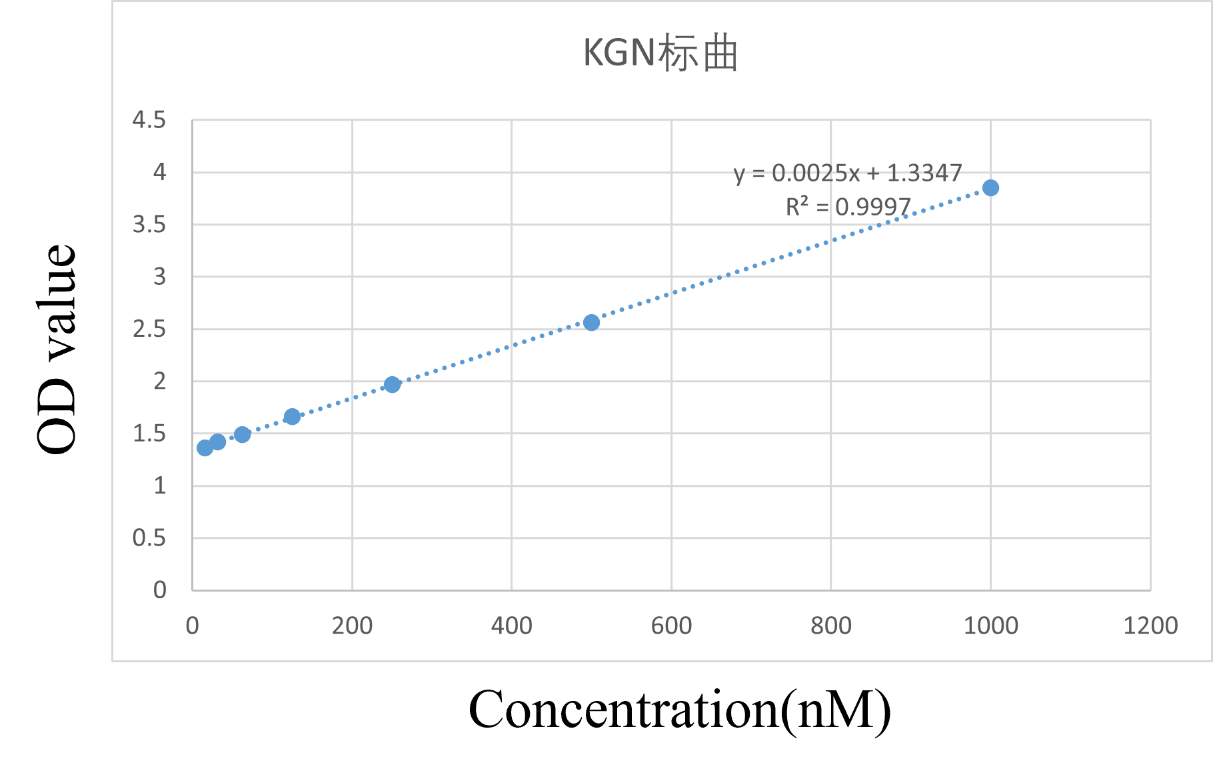


**Figure S5:** **KGN standard concentration curve.**


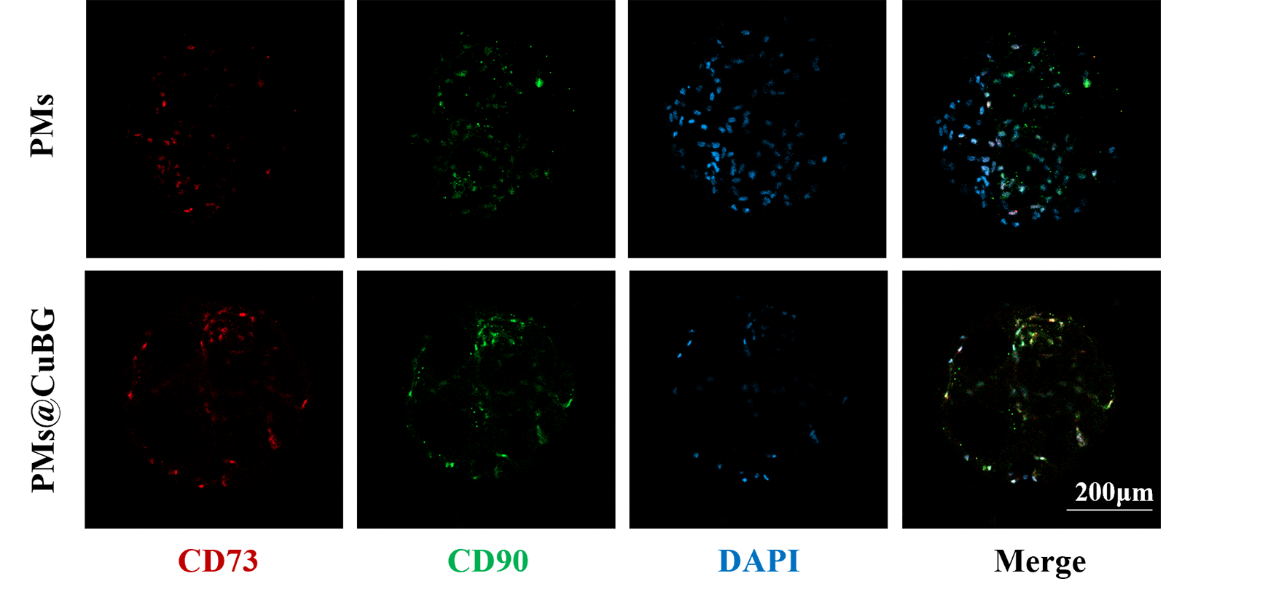


**Figure S6:** **Immunofluorescence staining of CD73 and CD90 after injection of microspheres into the knee joint of rats for 7 days.**


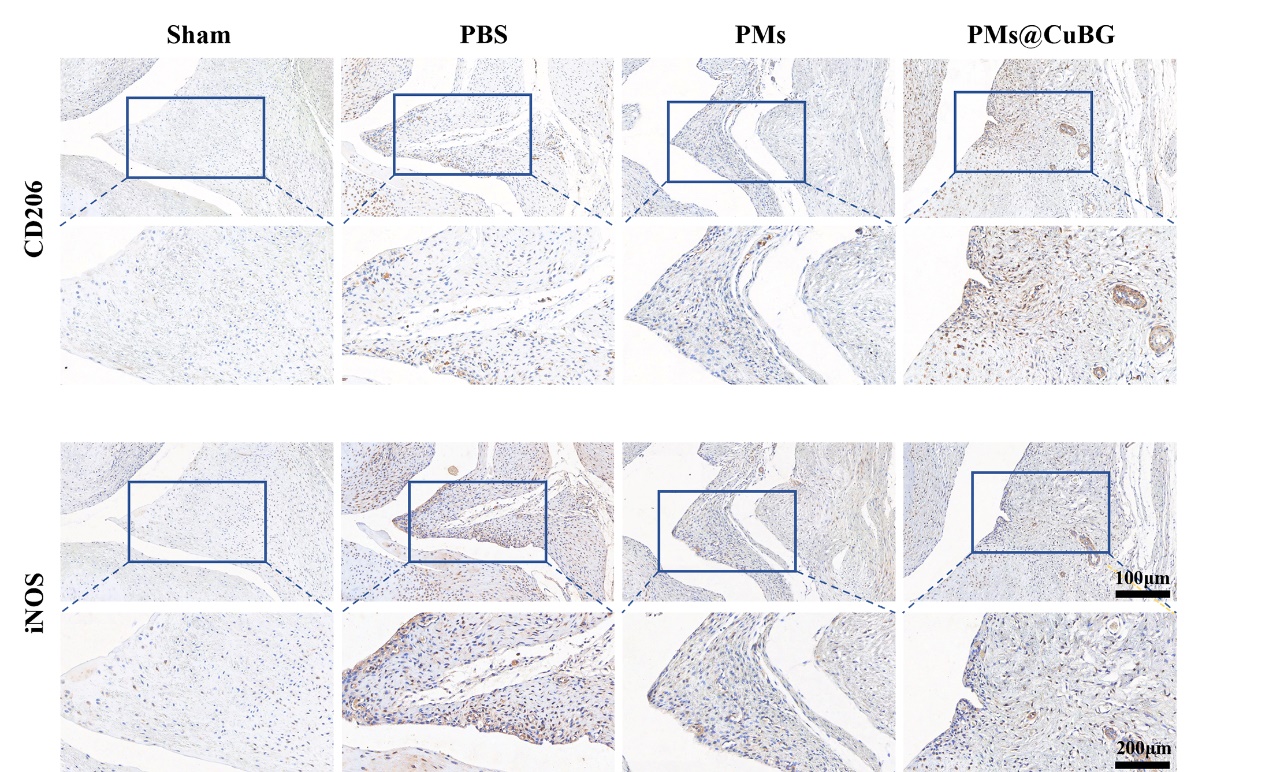


**Figure S7:** **CD206 and iNOS immunohistochemical staining image of the Section.**
